# Supplementary material for: KSHV vIL-6 Enhances Inflammatory Responses by Epigenetic Reprogramming
Source: bioRxiv. 2023 Jul 23:2023.06.25.546454. Preprint. [Version 2] doi: 10.1101/2023.06.25.546454 (PMC10370004; doi:10.1101/2023.06.25.546454)
Supplement: Supplement 1 [file NIHPP2023.06.25.546454v2-supplement-1.pdf]

bioRxiv preprint doi: https://doi.org/10.1101/2023.06.25.546454; this version posted July 23, 2023. The copyright holder for this preprint (which was not certified by peer review) is the author/funder, who has granted bioRxiv a license to display the preprint in perpetuity. It is made available under aCC-BY 4.0 International license.

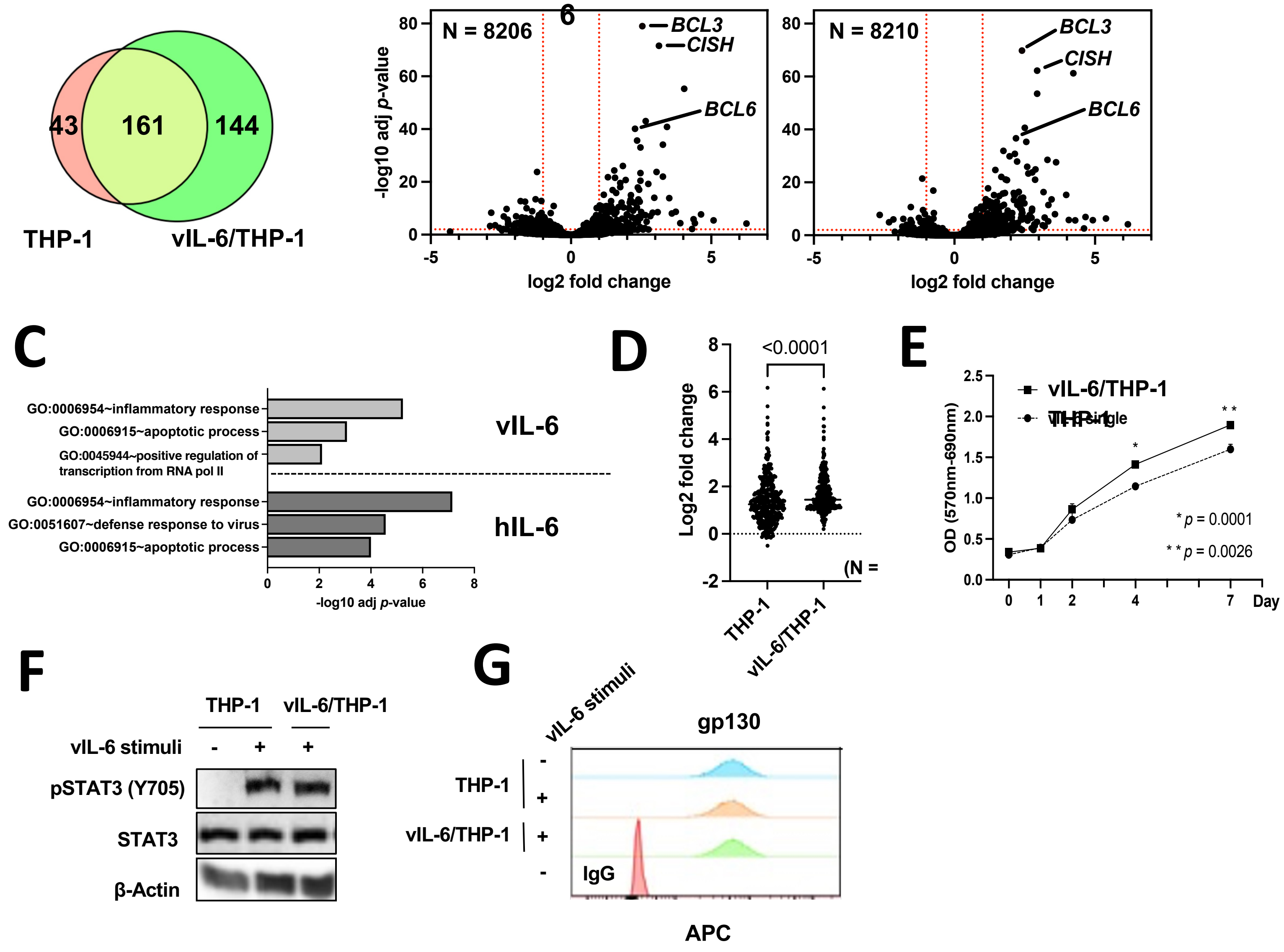

SFig.1 hIL-6 is a functional homolog of vIL-6 (A) The number of up-regulated genes (log2 fold change >1, adj *p*-value < 0.01) after hIL-6 stimulation. Red circle represents THP-1 cells and green circle represents vIL-6/THP-1 cells. **(B)** Individual gene expression in THP-1 cells with vIL-6 stimulation (left, N = 8206) and hIL-6 stimulation (right, N = 8210). Representative gene names were labelled adjacent to dots. The red dashed line indicated log2 fold change = ±1 (vertical) and -log10 adj p-value = 2 (horizontal). **(C)** KEGG pathway analysis performed on up-regulated genes (log2 fold change >1, adj *p*-value < 0.01) in THP-1 cells with vIL-6 and hIL-6 stimulation. The result showed the top three pathways each. **(D)** Individual up-regulated gene expression (N = 348) in parent THP-1 and vIL-6/THP-1 cells after hIL-6 stimulation. Data were analyzed using Wilcoxon matched-pairs signed ranked test and shown as median. **(E)** Measurement of cell proliferation with MTT assays. 1 X 10^4 THP-1 or vIL-6/THP-1 cells were cultured in triplicate in a 96 well plate. vIL-6 was added to vIL-6/THP-1 cells every other day. OD (570-690nm) was measured on day 0,1,2,4 and 7. Data were analyzed using unpaired Student's *t* test and shown as mean ± SD. **(F)** Immunoblotting with antibodies directed against STAT3, pshopho-STAT3 (Y705) and β-Actin (loading control) protein in THP-1 and vIL6/THP-1 cells. (G) FACS analysis showing the gp130 expression on cell surface.

SFig.1

bioRxiv preprint doi: https://doi.org/10.1101/2023.06.25.546454; this version posted July 23, 2023. The copyright holder for this preprint (which was not certified by peer review) is the author/funder, who has granted bioRxiv a license to display the preprint in perpetuity. It is made available under aCC-BY 4.0 International license.

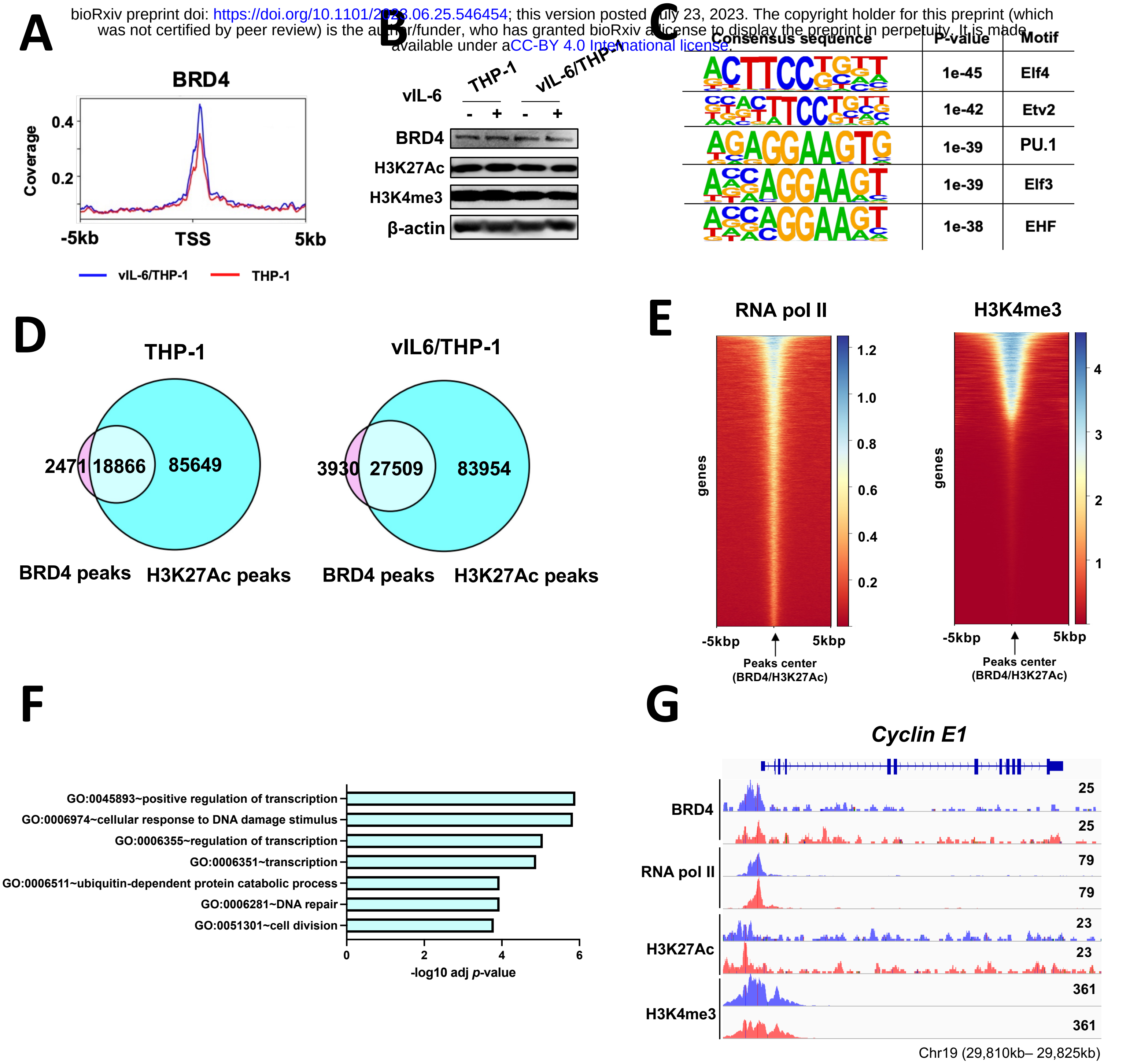

SFig.2 Prolonged vIL-6 exposure enhances the association of BRD4 and H3K27Ac (A) BRD4 CUT &RUN signals in ±5kbp windows around the transcription start sites (TSS) of up-regulated genes in vIL-6/THP-1 cells (N = 303). **(B)** BRD4, H3K27Ac and H3K4me3 protein expression before and after vIL-6 stimulation in parental THP-1 and vIL-6/THP-1 cells. **(C)** DNA binding motif analysis of new BRD4 accumulation sites in vIL-6/THP-1 cells. Images were drawn by findMotif (HOMER). **(D)** The number of BRD4 and H3K27Ac peaks and their association in parental THP-1 and vIL-6/THP-1 cells. The overlapping peaks were extracted using mergepeaks (HOMER). **(E)** RNA pol II and H3K4me3 CUT &RUN signals in ±5kbp windows around the center of BRD4 and H3K27Ac peaks. **(F)** KEGG pathway analysis performed on genes at BRD4 and H3K27 overlapping peaks in promoter regions in vIL-6/THP-1 cells. Results are presented in descending order. **(G)** BRD4, RNA pol II, H3K27Ac and H3K4me3 enrichment in the *Cyclin E1* promoter region in parental THP-1 cells (pink) and vIL-6/THP-1 cells (blue). The peaks were visualized by importing the BAM files into the Integrative Genomics Viewer (IGV).

SFig.2

bioRxiv preprint doi: https://doi.org/10.1101/2023.06.25.546454; this version posted July 23, 2023. The copyright holder for this preprint (which was not certified by peer review) is the author/funder, who has granted bioRxiv a license to display the preprint in perpetuity. It is made available under aCC-BY 4.0 International license.

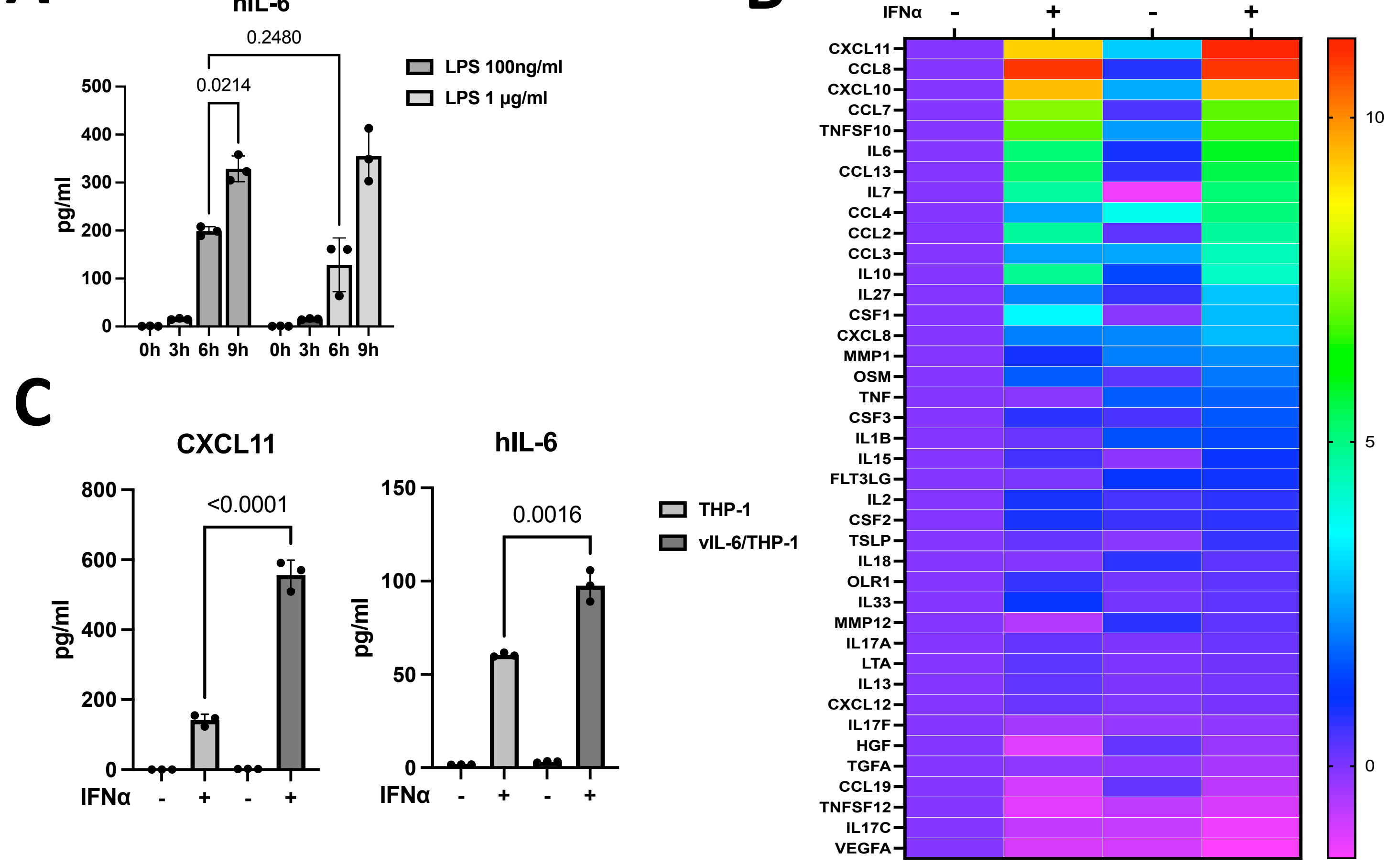

SFig.3 Inflammatory response to IFNα after vIL-6 prolonged exposure (A) hIL-6 production in THP-1 cells by LPS stimulation. THP-1 cells was incubated with LPS 100ng/ml or 1μg/ml for various time periods. Supernatants were harvested and incubated in triplicate in ELISA plate coated with hIL-6 antibody, Human IL-6 Uncoated ELISA kit (Invitrogen) was then used to evaluate the hIL-6 production by following the manufacturer's guideline. The protein binding measured as OD values at 450nm was shown. Results are presented as mean percentage viability ±SD ($n = 3$ samples/group). Data was analyzed by a one-way ANOVA test. **(B)** Heatmap showing the results of Olink® Target 48 Cytokine panel . IFNα (100ng/ml) was added to parent THP-1 cells or vil-6/THP-1 cells for 6 hours. Cytokine production in untreated THP-1 cells was set as 1 and $\log_2$ fold activation relative to untreated cells are shown. Samples were prepared in triplicate and the mean value were shown. **(C)** Inflammatory cytokine production determined by Olink proximity extension assay. Data was analyzed using two-sided unpaired Student's *t* test and shown as mean ± SD.

SFig.3

bioRxiv preprint doi: https://doi.org/10.1101/2023.06.25.546454; this version posted July 23, 2023. The copyright holder for this preprint (which was not certified by peer review) is the author/funder, who has granted bioRxiv a license to display the preprint in perpetuity. It is made available under aCC-BY 4.0 International license.

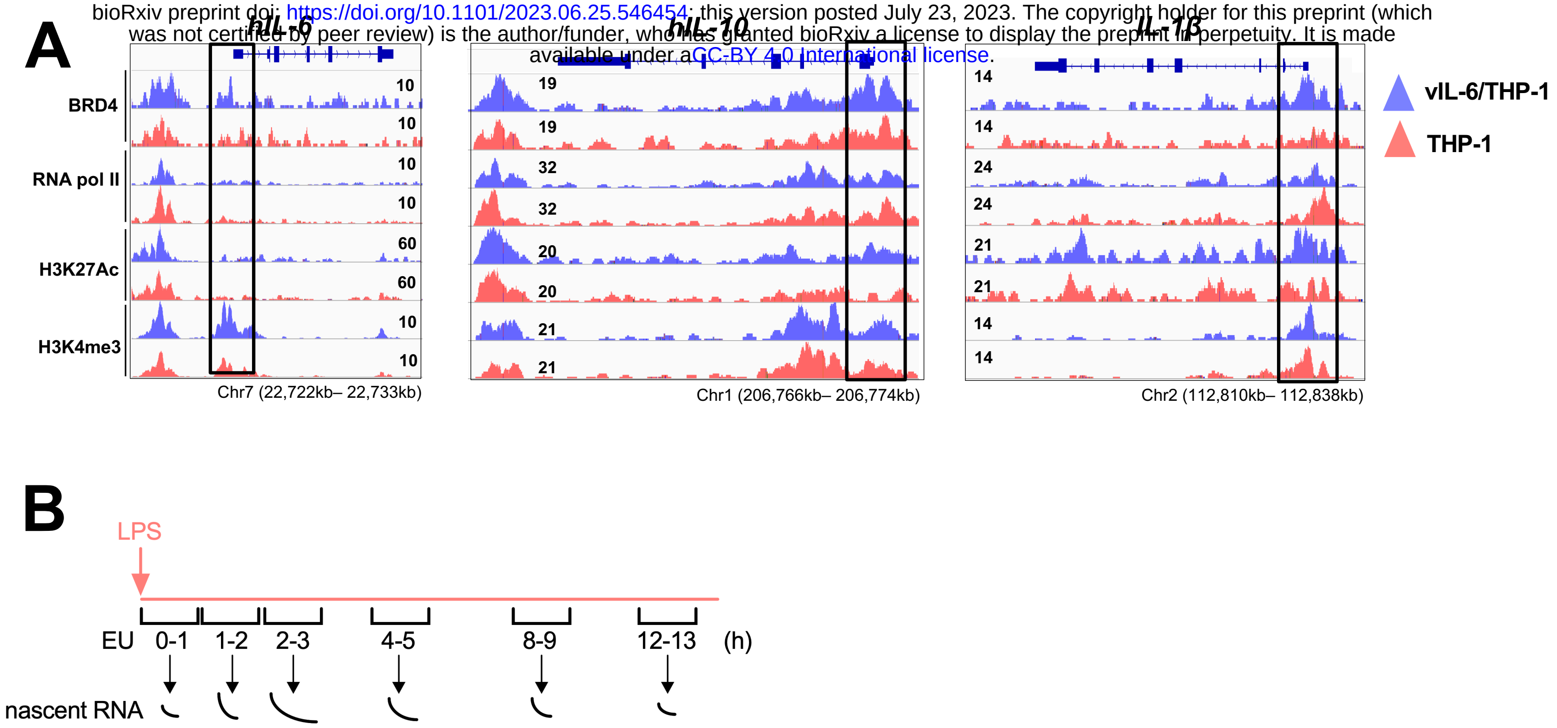

SFig.4 BRD4 enrichment in the promoter region of inflammatory genes (A) BRD4, RNA pol II, H3K27Ac and H3K4me3 enrichment in hIL-6, IL-10, IL-1β promoter region in parental THP-1 cells (pink) and vIL-6/THP-1 cells (blue). The promoter region is enclosed by a black line. Each CUT&RUN peak was visualized by importing the BAM files into Integrative Genomics Viewer (IGV). **(B)** Schematic diagram of nascent RNA labeling after LPS stimulation. LPS were added to culture medium in parental THP-1 and vIL-6/THP-1 cells and cells were incubated with EU for 1 hour in 0, 1, 2, 4 8, 12 h LPS stimulation.

SFig.4

bioRxiv preprint doi: https://doi.org/10.1101/2023.06.25.546454; this version posted July 23, 2023. The copyright holder for this preprint (which was not certified by peer review) is the author/funder, who has granted bioRxiv a license to display the preprint in perpetuity. It is made available under aCC-BY 4.0 International license.

Table. S1. Primer used for RT-qPCR and Chip-qPCR

**RT-qPCR primer**		
hIL-6	Forward	GGTACATCCTCGACGGCATCT
	Reverse	GTGCCTCTTTGCTGCTTT
IL-10	Forward	GGTTGCCAAGCCTTGTCTGA
	Reverse	AGGGAGTTCACATGCGCCT
IL-1β	Forward	AAACAGATGAAGTGCTCCTTCCAGG
	Reverse	TGGAGAACACCACTTGTTGCTCCA
18S rRNA	Forward	TTCGAACGTCTGCCCTATCAA
	Reverse	ATGGTAGGCACGGCGACTA
**Chip-qPCR primer**		
hIL-6 promoter	Forward	AACTTGGTTCAGGAGTGTCTAC
	Reverse	AGGATGGCTGGATGGTTTC
IL-10 promoter	Forward	GGAGATCTCGAAGCATGTTAGG
	Reverse	GGACAGAGAGGTGAAGGTCTA
IL-1β promoter	Forward	CAGCCAATCTTCATTGCTCAAG
	Reverse	CATACACACAAAGAGGCAGAGA
